# Developing reliability centered maintenance in automotive robotic welding machines for a tier 1 supplier

**DOI:** 10.3389/frobt.2025.1620370

**Published:** 2025-09-09

**Authors:** H. T. O. Alaka, K. Mpofu, B. I. Ramatsetse, T. A. Adegbola, M. O. Adeoti

**Affiliations:** 1 Department of Industrial Engineering, Tshwane University of Technology, Pretoria, South Africa; 2 Department of Industrial Engineering, Witwatersrand University, Johannesburg, South Africa; 3 Department of Mechanical and Mechatronics Engineering, Tshwane University of Technology, Pretoria, South Africa

**Keywords:** reliability, maintenance, robotic welding, reliability centred maintenance, optimising

## Abstract

The study highlights the effectiveness of FMEA in robotic spot-welding operations, providing a systematic approach to enhancing performance in an automotive assembly line. Robotic welding industries depend on mechanized, programmable tools to automate welding processes, ensuring efficiency, reliability, and effective material handling. In the automotive sector, Tier 1 suppliers utilize robotic welding machines to produce high volumes of welded assemblies, with daily output exceeding 450 units. However, frequent equipment downtime due to maintenance challenges disrupts productivity and impacts customer satisfaction. This study aims to develop a Reliability-Centered Maintenance (RCM) approach for robotic welding industries, optimizing machine uptime, enhancing product quality, and reducing financial losses caused by unexpected failures. A 3-year dataset was analysed to identify the primary causes of downtime and their associated costs. Failure Modes and Effects Analysis (FMEA) was applied to assess failure modes, determine root causes, and calculate Risk Priority Numbers (RPNs), thereby formulating corrective actions to mitigate recurring failures and enhance operational efficiency. Findings revealed that maintenance-related issues accounted for 79% of total downtime, resulting in financial losses of R2,281,508.82 over 3 years. The application of FMEA provided a structured framework for prioritizing critical failure modes and implementing targeted corrective measures to reduce downtime and enhance overall reliability. To sustain high productivity and quality, it is recommended that robotic welding industries adopt proactive maintenance strategies based on FMEA findings. Regular monitoring, predictive maintenance, and workforce training will help minimize machine failures and optimize operational efficiency.

## 1 Introduction

Robotic welding processes have become increasingly important in recent years. It is important for the machinery involved in this operation to be available all the time to ensure a quality weld. The equipment directly and indirectly involved in the production line must be uninterrupted. RCM has long been used in the aviation industry and military to ensure the reliability of assets (Refinery and Dharmaraj, 2019).

RCM has had its methodology applied in various sectors over the years, besides the previously mentioned aviation and military industries. The United States military saw a strong benefit to using RCM as a reliable method of combating robot failures on the frontlines (MSG-3, 2018). The implementation of the RCM’s methodology ended up being a cost-effective method that has been used by the military for decades. In the aviation sector, RCM was described as an optimum maintenance process used to meet all the maintenance requirements, which was formulated due to the first-generation jet aircraft’s crash rate (Bugaja et al., 2019). In the Walt Disney Company, the RCM program was brought about mainly to ensure the safety of the company’s machinery and proper scheduling of its maintenance. The implementation of the methodology turned out to be a major cost-saving approach for the company (Anton and Yoshino, 2003). Lastly, in research conducted to fix the automation maintenance problem in the ArcelorMittal Hot Strip Mill production line, the RCM approach was customised to the organisation’s benefit by systematically developing the basics of RCM principles and tools such that success is demonstrated in the shortest time, given the small number of resources available (Fouché, 2015).

In a more recent application of RCM methodology, the FMECA approach was systematically applied to a 1600T press experiencing unplanned downtime and inconsistent production line productivity in an organisation called Algal+ (Ghemari et al., 2024). The FMECA approach implemented significantly contributed to optimizing the reliability and equipment availability, thereby reducing unplanned downtime and enhancing operational efficiency through data-driven measures focused on addressing the most critical risks experienced on the press.

Therefore, the automotive robotic welding sector can be compared to the airline sector, due to its functionality. The robotic welding industry’s productivity relies mainly on high equipment reliability and availability. The research problem faced on the production line goes as thus: “The current equipment availability stands between 70% and 72%, which is far from the required 97% needed to meet the production needs for the robotic welding machines to supply demand as a Tier 1 company to an Original Equipment Manufacturing”. RCM covers all the different types of maintenance, and it can be applied to any type of industry using any kind of machine (Fouché, 2015). The skilled-employee shortage is a major problem for industrial manufacturing. The American Welding Society specified that 40% of manufacturing companies declined new contracts because not enough skilled workers were available (Halle, 2019). A lack of flexibility is cited as the major reason 90% of all manufacturing companies do not have robotic systems. Therefore, the proper maintenance of the equipment is necessary, and a system needs to be developed to improve the robotic machine’s availability.

Following the problem statement, the study aims to develop an RCM approach in automotive robotic welding machines for a tier 1 supplier to improve the availability of the welding machines that which can lead to an increase in productivity and profitability. The research objectives to achieve the research questions includes, identifying a wide range understanding of the RCM process, concepts, elements, developing strategies, and successes, advantages, and disadvantages as a complete maintenance system, determining the current availability of the equipment on the production line to ensure the development of RCM for robotic welding machines, evaluating different maintenance approaches and compare them with the traditional maintenance processes such as reactive and preventive maintenance applied in the company machine/equipment availability improvement, investigating the RCM development success stories to evaluate all the different approaches the organisations used to ensure the RCM’s successful development and, lastly, developing a tailor-made RCM through the creation of FMEA to improve robotic welding machines thereby increasing machine availability.

**FIGURE 1:**
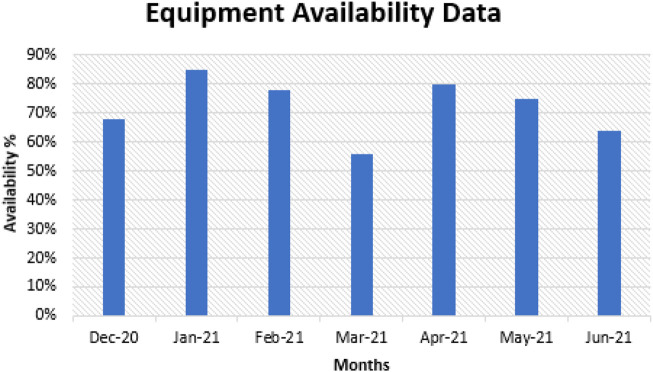
Equipment availability data (Tier 1 company to an OEM’s data).

The world-class target of equipment/assets availability in a production line is 97% (Ahmad and Benson, 2007). The monthly availabilities for the period December 2021 to June 2022 are shown in Figure 1. As shown in the figure, there is no consistent pattern in the availability patterns.

Due to the inconsistencies in the production lines' performance, the deduced problem statement goes as follows: The perception of RCM is a comprehensive process that is very time and resource-consuming; therefore, what are the difficulties in developing RCM successfully in the robotic welding industries?

The study was created using a model based on certain characteristics and data-collection strategies. These methods for maintenance problems aim at enhancing the effectiveness of machines to eventually improve productivity. These include Reliability-Centred Maintenance, Component System Selection and Data Collection, Logic Tree Analysis (LTA), and Criticality Analysis. These strategies were used to create an adopted FMEA model based on the following characteristics and data collection strategies. These models to be emphasized would be the principal maintenance strategies, rather than being applied autonomously, combined to take advantage of their corresponding strengths to take full advantage of the robotic welding machines’ reliability while minimizing life-cycle costs.

The FMECA is an analysis tool widely used and accepted to improve maintenance practices in most process industries (Pancholi and Bhatt, 2018). Before focusing on the study’s design and methodology, the advantages or importance of developing RCM are: RCM has the potential to be the most efficient maintenance strategy when developed, applied, and maintained correctly, therefore leading to reduced maintenance scheduling on the production line; comprehensive equipment analysis will reduce maintenance costs by eliminating unnecessary asset maintenance, and; RCM improved the reliability of equipment, thereby causing the risk of failure of equipment on the line to be significantly reduced.

Although there are many pros to developing the RCM plan for any process, there are also some cons, including the high cost of initially starting the RCM process and the time it takes for the results of the newly developed process to be seen.

Therefore, to effectively ensure the maintainability and reliability of machinery on the production line, according to a guideline set by SAE, the RCM method requires analysis of equipment using the FMEA, using the maintenance costs or downtime report data to determine the equipment’s status, then application of processes to achieve the purpose of the action and analysis of data post-implementation phase to ensure that the newly implemented system is functional (SAE JA1011, 2022). Therefore, to achieve effective implementation using data, certain questions need to be answered satisfactorily and in the listed sequence to determine if an organization is following RCM standards: these include, What are the functions and associated desired standards of performance of the asset in its present operating context (functions)?In what ways can it fail to fulfil its functions (functional failures)? What causes each functional failure (failure modes)? What happens when each failure occurs (failure effects)?In what way does each failure matter (failure consequences)? What should be done to predict or prevent each failure (proactive tasks and task intervals)? And what should be done if a suitable proactive task cannot be found (default actions)?

Analysing the pros and cons of RCM, the advantages are greater than the disadvantages. Therefore, this system can be a great achievement in any organisation.

**FIGURE 2:**
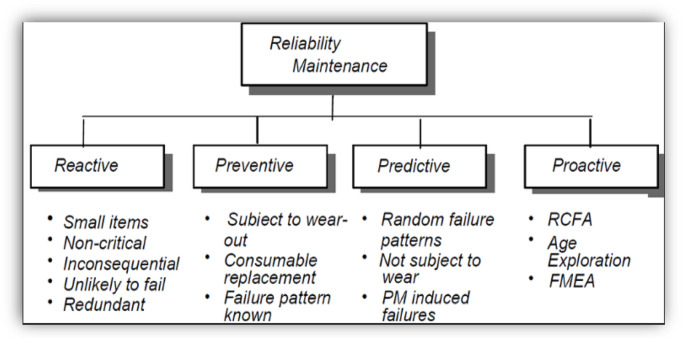
Components of an RCM programme (Okwuobi et al., 2018).

Furthermore, RCM is thus a combination of PM scheduling, corrective/unplanned/reactive maintenance practices, and first-line tasks/proactive maintenance (Fouché, 2015). Figure 2 shows the components of the RCM programme (Okwuobi et al., 2018).

Equipment reliability and its importance it is rarely questioned. Improved equipment reliability can reduce maintenance costs, minimize opportunity costs associated with downtime, and ensure products are not produced outside of acceptable specifications. Understanding how programs, procedures, and equipment are improving reliability at a facility is vital to understanding overall program effectiveness and what steps could best be taken to further improve equipment reliability. It is for this reason that, although often overlooked, the trending of an equipment reliability program is an important part of facility management.

This study, therefore, aims to develop a Reliability-Centered Maintenance (RCM) approach for robotic welding industries to optimize machine uptime, improve product quality, and minimize financial losses caused by unexpected failures.

Table 1 shows a comparative summary table highlighting the differences between FMEA in RCM and related approaches carried out by other researchers, emphasizing the contribution and novelty of integrating FMEA specifically into RCM, especially relevant to automotive robotic systems or industrial settings.

**TABLE 1:** Comparative summary table highlighting differences between the proposed method and related works.

Study	Sample	Method	Reliability	Scope
Ramli and Arffin (2012)	All Class A, B, and C equipment in an automotive manufacturing company	RCM and FMEA	N/A	Reduction of the operator’s workload through RCM implementation
Kolte and Dabade (2017)	Cylinder block manufacturing line in an automobile engineering company	Preventive Maintenance (PM)	Developing a PM frequency for failures	Improving the availability of critical equipment
Yavuz et al. (2019)	Packaging machine	RCM	N/A	Effect of RCM on OEE of packaging machines
Zeinalnezhad et al. (2020)	Critical Success Factors (oil and gas plant)	Expert Group discussion and Fuzzy Analytic Network Process	Calculation and Matrices of Eigenvectors	Improve plant reliability at a lower cost
Suryono and Rosyidi (2018)	Laser machine in Filling Lithos at PT X	RCM, RPN	Decreased downtime from 1273,76 min–17 min using FMEA.	Reduction of downtime

## 2 Methodology

The data was collected from the robotic cell it focuses on, as well as the research methodology using Failure Modes and Effects Analysis (FMEA) and Linear Regression (LR). The study demonstrates the data collection method direction in the determination of the problems connected with automotive robotic welding machines for a tier 1 supplier in the development of RCM.

### 2.1 Data collection

#### 2.1.1 Downtime

Data was analysed for the downtime experienced by the robots used to perform the automated welding process on the production line and to identify areas where the number of stoppages can be reduced and improve the overall efficiency.

**FIGURE 3:**
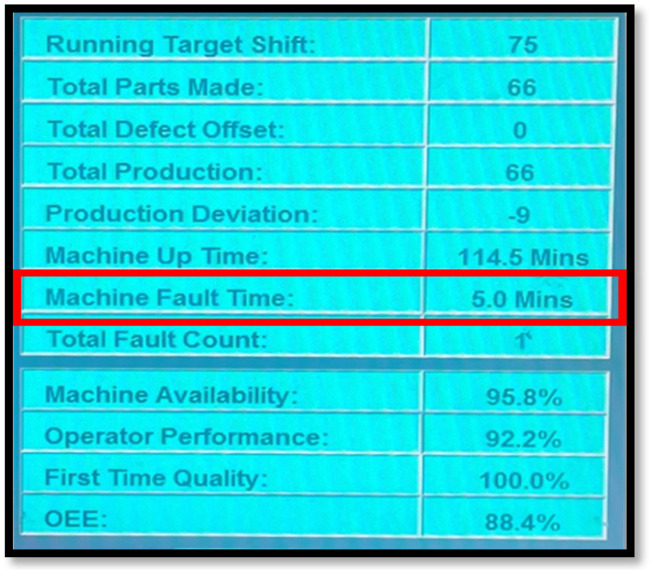
Machine fault time/downtime identification.

Figure 3 shows the HMI screen of the robotic welding machine, showing the total fault time or downtime experienced during the cause of the shift.

The data was calculated by summing up the total number of downtimes recorded in minutes during the month, as shown in Equation 1, and dividing by the actual number of working days, as shown in Equation 2. The formula used is as follows:

$$\sum \left(x\right)=x_{1}+x_{2}+.....+x_{12}$$ (1)

where $x_{1}$ is the average downtime per month in minutes.

$$Average\;downtime/month=\frac{y_{1}+y_{2}+.....+y_{n}}{n}$$ (2)

Where y is the downtimes occurring per day, and n is the total working days in the month.

Equation 3 represents the formula for calculating the average downtime in a year, which is (12 months):

$$Average=\frac{x_{1}+x_{2}+.....+x_{12}}{12-number\;of\;months\;of\;no\;planned\;production}$$ (3)

#### 2.1.2 Uptime

By monitoring uptime, you can identify potential issues or areas for improvement that can help reduce downtime and increase productivity. This could ultimately lead to reduced prices and higher-quality goods.

To calculate the uptime in robotic systems, there is a need to know the total operating time and the downtime of the system during the specified period. Once these two pieces of information have been gathered, the commencement of uptime calculation can be done using the formula in Equation 4:

Uptime=Total operating time−downtime (4)

**FIGURE 4:**
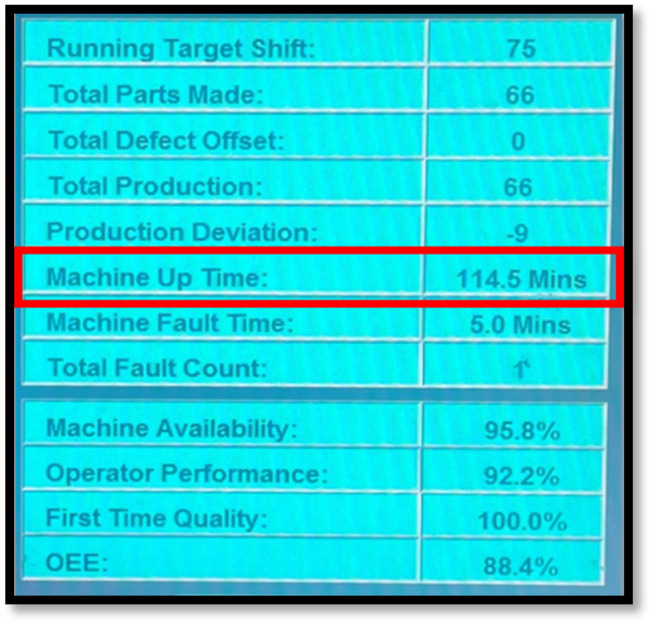
Machine uptime identification.

Using information from uptime analysis, one can prioritize equipment repairs or replacements based on which machines are causing the most downtime. The uptime data is obtained from the HMI screen data. The HMI data is automatically generated by calculating the actual time the machine is in operation (Figure 4).

#### 2.1.3 Breakdown

**FIGURE 5:**
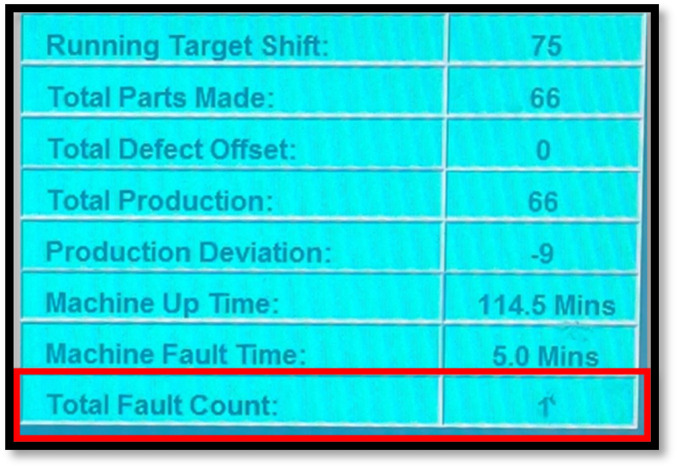
Machine fault time identification.

Conducting a breakdown analysis on robotic welding machines can help improve machine performance, reduce downtime and costs, and support continuous improvement efforts. The data collected for the breakdown analysis was also obtained from the Human-Machine Interface (HMI) screen and input manually on the breakdown input spreadsheet. Figure 5 shows the HMI screen data in which the “Machine Fault Time” values are collected. The breakdown is automatically recorded after every stop experienced by the cell regarding maintenance.

#### 2.1.4 Mean time between failure (MTBF) of the CM #4 robotic weld cell

The Mean Time Between Failure (MTBF), Mean Time to Repair (MTTR), and failure rates of the individual components evaluated the distribution and reliability of each identified system component (Atikpakpa et al., 2016). The calculation of the downtime, uptime, and breakdowns experienced by the robotic welding machine is focused on, and the data obtained is used to effectively calculate the MTBF of the weld cell per year for the 3 years of data analysed.

For the study, the formula used to calculate the MTBF for the robotic welding machine, including each 12-h shift (morning and night shift) for the 2020, 2021, and 2022 production data gathered, Equation 5 for 2020 data, Equation 6 for 2021 data, and Equation 7 for 2022 data, is:

MTBFα=If βα; xα60; xαβα60  (5)

where α is the morning shift, ${\;\beta }_{\alpha }$ is breakdown per month (morning shift), and $x_{\alpha }$ is the total uptime per month (morning shift).

MTBFγ=If βγ; xγ60; xγβγ60  (6)

where γ is the night shift, ${\;\beta }_{\gamma }$ is the breakdown per month (night shift), and $x_{\gamma }$ is the total uptime per month (night shift).

To get the total monthly MTBF, the previously calculated total uptime time per month and total breakdown time summed per month for each year of data collected are as follows.

MTBFT=If βT; xT60; xTβT60  (7)

Where ${MTBF}_{T}$ is the total MTBF for the month, ${\;\beta }_{T}$ is the total breakdown per month, and $x_{T}$ = total uptime per month.

Then, the average MTBF was calculated using Equation 8.

$${MTBF}_{\mu }=\frac{{MTBF}_{T1}+{MTBF}_{T2}+{MTBF}_{T3}+...+{MTBF}_{T\infty }}{Number\;of\;Productive\;Month}$$ (8)

where ${MTBF}_{\mu }$ = Average MTBF for the year.

### 2.2 FMEA

In this research, FMEA is developed and proposed to mitigate the risks of failures in the weld cell. The failures that occurred in the machine were identified through the data recorded by maintenance after corrections were implemented in the form of a reactive maintenance plan.

The failure modes stated are gathered from the current maintenance data as the main contributing factors affecting the availability of the station. Each failure mode is based only on the maintenance factor of the robotic welding machine. The RPN of the failure modes from the previous maintenance data collected in the past 3 years is calculated as the product of severity (S), occurrence (O), and detection (D) (Ramli and Arffin, 2012). In identifying the RPN Matrix, the occurrence, severity, and detection of failures in the development of the FMEA were determined using various matrices.

### 2.3 Linear regression model for comprehensive method

Alternative models that could be employed include Random Forests and Decision Trees, both of which can be computationally demanding and need extremely non-linear data to be collected. As a result, the method is less interpretable than linear regression. An additional model is the Lasso and Ridge Regression. By introducing penalties to avoid overfitting and manage multicollinearity, this model goes beyond linear regression. Although it can zero out features and yet assume linear correlations, it may lose important predictors. When model resilience needs to be improved but interpretability is still sought, this method works well. A rationale for employing the linear regression model is the limited quantity of maintenance data available for the study.

The Regression analysis model, as an RCM comprehensive approach, is known to be a statistical approach that is used to examine the relationships among variables. This regression analysis format, frequently used for the correlation study, is the linear regression model. The linear regression model is uncomplicated and straightforward, but also a very effective methodology used in the investigation of the association between a dependent variable and an independent variable (Nahatai et al., 2023).

The linear regression analysis model is also known as a mathematical statistical approach using data collected from a process for probing connections between variables used in several fields, including the financial side, manufacturing, behaviour, environmental science, sociology, and medicine, and many other institutions (Montgomery et al., 2021).

A model for improving MTBF and MTTR for various copper mine sites located in Chile, and it functions by inputting non-linear autoregressive exogenous variables using a vector mechanism and historic data collected (Curilem et al., 2014). These data collected from past occurrences helped with the calculation of an inert view of the failures occurring in the different equipment of the system and the assessment of different failure situations that could occur in the mines.

The mathematical formula for a linear regression model is defined in Equation 9 (Paolella, 2018),

y=ax+b+η (9)

Where **y** is the conditional variable, **a** is the regression intercept term, **b** = the regression slope coefficient, **x** is the independent variable, and ***η*** is the random variable is the error term in the model.

**FIGURE 6:**
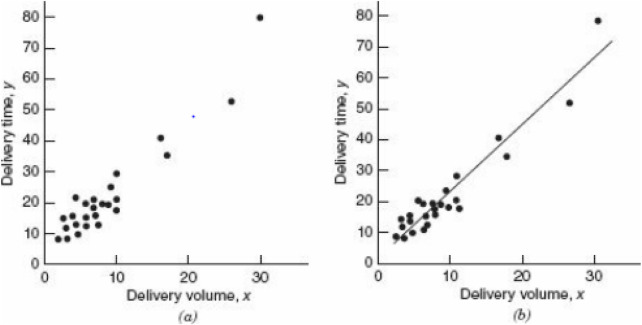
**(a)** Scatter diagram for delivery volume; **(b)** Straight-line relationship between delivery time and delivery volume (Nahatai et al., 2023).

Another formula for the linear regression is represented (Nahatai et al., 2023) in Figure 6 shows an example of a linear regression model, where the first graph shows a scatter diagram and the second shows the relationship between the scatter diagram and the straight-line using Equation 10 to be derived:

$$f\left(x\right)=\beta_{0}+\beta_{1}x+\varepsilon$$ (10)

Where f(x) is the dependent variable, ε is the random component, ${\;\beta }_{0}$ is breakdown per month (morning shift), and where x Is the average downtime per month in minutes

## 3 Results and discussion

Analysis was conducted on the availability of the robot welding machine used as a case study, the production values, the cost effect of downtime, linear regression effects in the 3 different years of data collected, a cause-and-effect diagram, and developing an FMEA for the maintenance team.

### 3.1 Relationship between availability and production

It is important to analyse the relationship between availability and production to be able to determine the effects of breakdown on the total amount of production. Therefore, the optimisation of equipment’s productivity or availability is complicated when there is a lack of information through data collection and analysis (El-Thalji and Liyanage, 2012).

**FIGURE 7:**
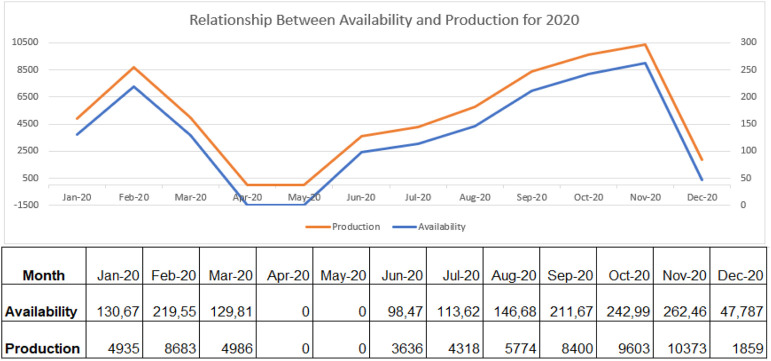
Relationship between availability and production for 2020.

**FIGURE 8:**
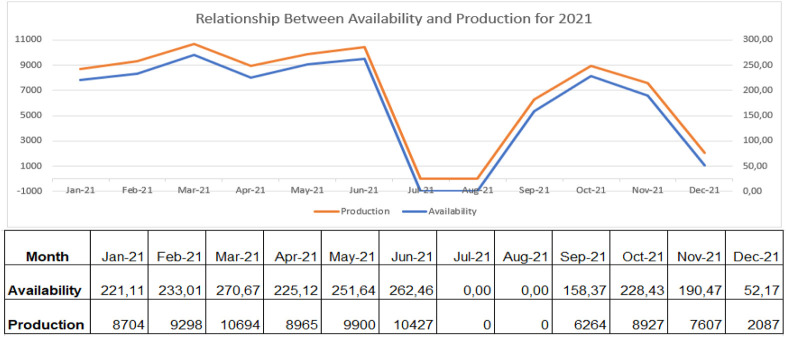
Relationship between availability and production for 2021.

**FIGURE 9:**
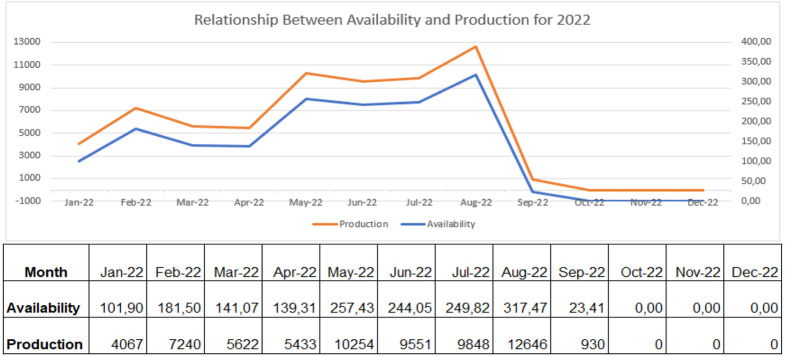
Relationship between availability and production for 2022.

From the above analysis, Figures 7–9 shows the relationship between availability and production on the robotic weld cell, focused on the 3 years of data collected for 2020, 2021, and 2022, respectively.

Consequently, if the welding station has all the required resources for production available, the machine can generate parts extremely well. Through the analysis of Figures 7–9, the availability of machines and production generated by the robot trail each other. The key objective of the research is, consequently, to tackle the reasons affecting the availability of machines and their effects on the process.

### 3.2 Analysis of downtime failures

In the analysis of downtime failures occurring in the robotic welding machine, it was determined that there is an immediate correlation connecting availability and production in the machine, as shown in the methodology. Therefore, the downtime justified additional analysis. The major contributing factors affecting downtime were found as follows:

- Man: Consisting of the period it takes when production operators execute production-related jobs like electrode changes, production recording, and 5S activities.
- Maintenance: This factor includes both mechanical and electrical breakdowns and repairs implemented on the welding cell.
- Material: Logistics personnel not having the required components on the welding cell, as they are required for production purposes.
- Quality: This factor consists of fixing dimensional problems on the welding fixture, which affects the final assembly.

Table 2 shows the different downtime contributors that affect the robotic welding machine according to the data gathered from the organisation from 2020 to 2022 in minutes.

**TABLE 2:** Major downtime contributors.

Maintenance	Awaiting material (logistics)	Man	Quality	Others
3,647	137	60	724	35
79%	3%	1%	16%	1%

**FIGURE 10:**
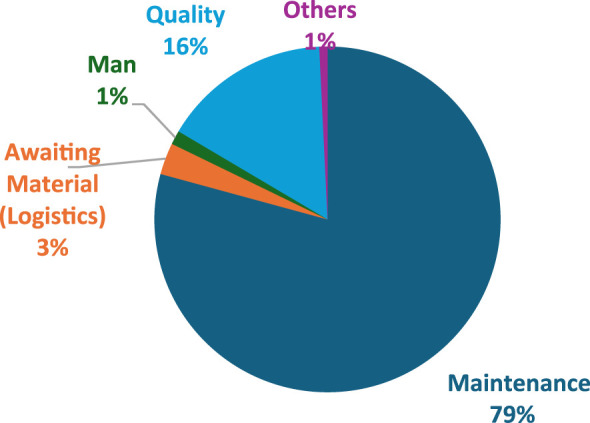
Factors affecting downtime.

Figure 10 represents the breakdown of factors affecting failures using a pie chart. It identifies maintenance downtimes as the major contributing factor, accounting for 79% of the failures.

Table 3 below shows the various types of maintenance failures that occur on the robotic welding machine. The data was extracted from a manually inputted breakdown action plan. All the data extracted equates to 79% of the maintenance failures. Also, tabling the faults or failures that occur in the machine and the total number of times that they appear on the line from 2020 to 2022.

**TABLE 3:** Maintenance failures and their occurrence.

Fault/Failures	Count
Clamp fault	60
Incomplete spot	15
Light curtain failure	60
Part sensor failure	35
Part stuck	20
Robot stuck	50
Spot out of position	15
Tip dressing	80
Water fault	95
Weak weld	30

**FIGURE 11:**
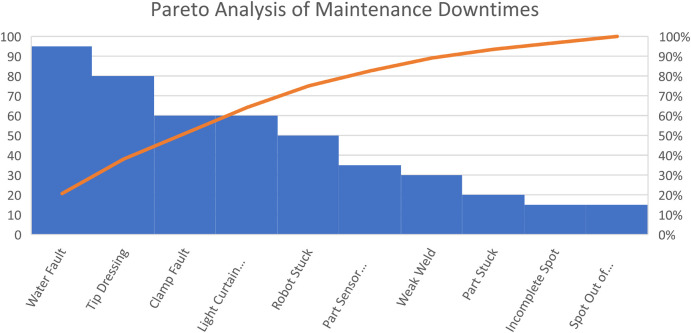
Pareto analysis of maintenance downtimes.

In Figure 11, a Pareto analysis was made using the data in Table 3. The chart shows that a water fault has the highest number of occurrences, happening 95 times in the 3 years. Also, the incomplete spot and spot out-of-position failures occurred 15 times during the 3 years of data collection.

Table 4 shows the failure mode and effects of all the listed maintenance downtimes analysed in the Pareto analysis for all different processes in the robotic welding operation.

**TABLE 4:** Failure effects and causes.

Process	Requirement	Failure mode	Failure effect	Failure cause
Part Loading	Correct part loaded	Incorrect/Incomplete part loaded	- Rejects and line stoppage. - Part failure at Customer	- Unskilled operator - Welding jig is not full proof to prevent incorrect loading
	Correct orientation of loading	Incorrect loading orientation		
Part Presence	The part should be detected on the fixture	Part presence sensor is not functioning effectively	The possibility of a missing component causing rejection on the customer line	- Part sensor malfunctioning - Broken sensor pins
Automated Clamping	Part clamping	Wrong Part clamping	- Scrap or reject - Downtime resulting in production loss	- A welding jig is not foolproof to prevent incorrect loading
		Clamp loose		The fixture clamp weight is loose
Robotic Welding Operation	Correct Number of spots on part (40 spots)	Position/quantity of spots does not match the 2D and 3D CAD drawing	- Scrap or reject - Downtime resulting in production loss. - Part failure at Customer	- Incorrect robot settings - As the robot teaching was not proper, the failure was identified
Robotic Welding Operation	Correct Weld Integrity	Weak spot	- The gap between the spots can create defective products. - Scrap or reject - Downtime resulting in production loss. - Part failure at Customer	- Incorrect welding parameter settings - Tip dressing was not correctly done
Robotic Welding Operation	No Spot Burr visible	Spot Burr	- Non-compliance with Customer Specification, part failure - If unchecked, this may break the part during the next operation. The body may become noisy at the customer end	- Tip dressing not correctly done. - High current flow at the welding spot due to incorrect parameter settings. - High welding time is observed, and there is a gap between parts
Robotic Welding Operation	No deep spot visible on the part	Deep Spot		
Robotic Welding Operation	Optimised electrode consumption	Excessive electrode consumption	Improper weld	Inadequate gas flow
Robotic Welding Operation	Spot should be round (by the spec of 4 mm diameter)	Spot size not in spec	- Scrap or reject - Downtime resulting in production loss	Tip dressing frequency is too low
Part Unloading	Parts are easy to offload	Part stuck on the fixture	- Downtime resulting in production loss	Build of splatter on fixture location pins

### 3.3 Cost analysis of downtime

The cost analysis of the downtimes experienced on the robotic weld cell being analysed was calculated for the 3 years (2020–2022) of data collected. According to Fore and Msipha (2010), the cost of the poor availability of equipment on a production line is directly proportional to the result of poor maintenance (Fore and Msipha, 2010). The sales value for each assembly sold to Motor Company of South Africa is R770.31.

In the analysis, 79% of downtime was caused by maintenance, summing up to R2,281,508.82 in the 3 years of data collected.

### 3.4 Results of the linear regression analysis

Linear regression was used in this research to show the connection between availability and production through the investigation of the MTBF derived from the calculations in the methodology (Chapter 2). The linear regression assessment establishes that there is an immediate association between improved availability and the welded assembly produced on the station. This emphasizes the element that any maintenance prospects that will increase availability in the welding station will directly increase the productivity of the station. Therefore, an application of refining availability by improving the current maintenance management system through RCM is valuable.

**FIGURE 12:**
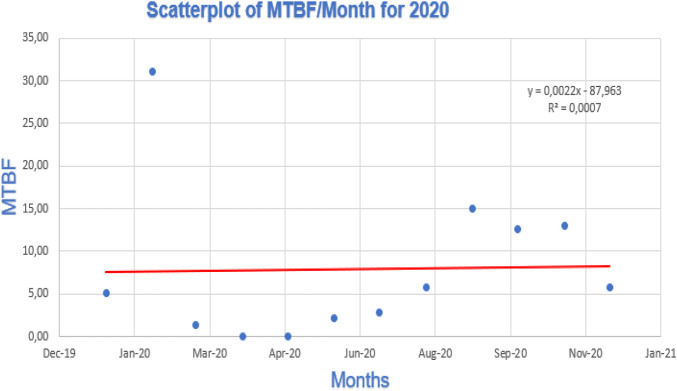
Linear regression for 2020.

**FIGURE 13:**
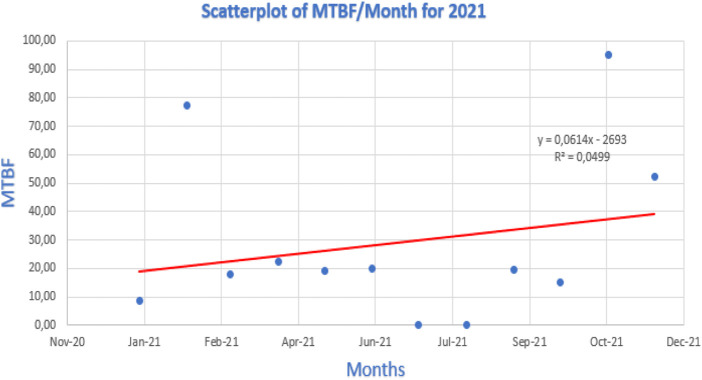
Linear regression for 2021.

**FIGURE 14:**
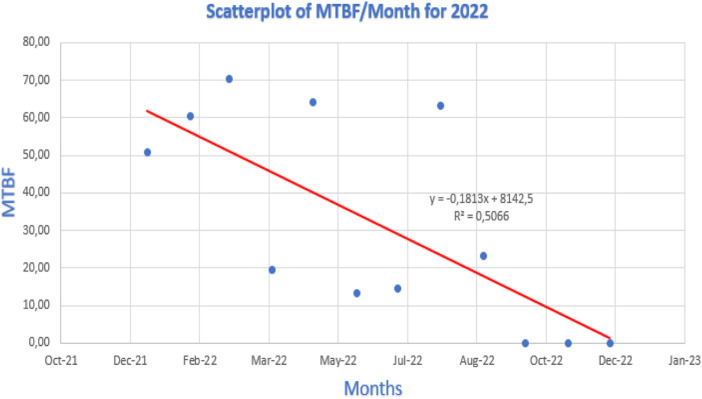
Linear regression for 2022.

Figures 12–14 show the linear regression scatterplot for MTBF/month for 2020, 2021, and 2022, respectively.

Figure 12 shows a great degree of scatter in the graph plotted. The high degree of scatter implies that there were poor levels of control occurring in 2022. Therefore, correcting this scatter by reducing the degree of scatter will ensure that there is improved availability of the welding station.

In 2020 and 2021, the gathered data and graph plotted in Figures 13, 14, respectively, revealed that there was a flatter trendline leaning positively, and the scatter plot was closer than in Figure 12. The degree of scatter in 2021 is more than the scatter in 2020. The different graphical representation shows an unstable maintenance strategy being followed by the maintenance department due to the 3-year data plotted having no trend in 2020, a positive trendline in 2021, and a negative trendline in 2022.

Due to the line’s observed flatness, the projected 2020 linear regression graph indicates there was no trend. In Figure 12, the trend line value was ***Y = 0,0022x - 87,963***, therefore, the MTBF linear regression analysis for a robotic welding machine may indicate that the variables under study are independent of one another and that there is no discernible association between them if the trend line is absent or flat.

According to the research findings for 2021, a robotic welding machine’s availability may rise if the MTBF linear regression analysis shows a positive trend line. A rising trend line shows that MTBF is becoming better over time. This implies that the machine experiences fewer failures, which leads to less downtime and more uptime. Finding the relationship between availability and MTBF was made possible in large part by statistical analysis. In Figure 13, the trend line value was ***Y = 0,0614x - 2693***.

An indicator of a system’s dependability is MTBF. The line dropping as it moves from the left to the right of the line observed was observed in the plotted 2021 linear regression graph as a negative trend. An MTBF linear regression study with a negative trend line shows that the system’s reliability is declining over time. This indicates that more failures occur with robotic welding equipment, which could result in more downtime and decreased availability.

When a machine’s availability is compromised, its ability to carry out its intended function for the required period is interfered with. This could lead to lost production, higher expenses, and even safety risks. To increase the machine’s reliability and keep it available, it is crucial to find the source of the negative trend line and take appropriate action. In Figure 14, the trend line value was ***Y = -0,1813x + 8142,5***.

## 4 Conclusion and recommendations

In this research, the tier 1 supplier will systematically discover, assess, and prioritize possible equipment failures before they happen by using FMEA to improve maintenance scheduling. Maintenance teams will change from reactive to proactive tactics by knowing which parts are most likely to fail and the consequences of those failures, as shown in Table 4. As a result, maintenance tasks become more focused and timelier, which lowers the possibility of unforeseen malfunctions that interrupt output.

Based on the Pareto Chart and the Failure Cause and Effect Table (Table 4), the organization can reduce production losses due to failures by up to 80% because of this strategic approach. The losses resulting from incorrect maintenance scheduling are significantly reduced, with R1,825,207.056 representing 80% of the losses before the RCM model. Overall equipment reliability and operational efficiency are improved by FMEA-driven maintenance scheduling, which reduces unscheduled downtime and concentrates resources where they are most required. In addition to maintaining output, fewer interruptions result in cheaper repairs and more productive workers.

The selection between FMEA and RCM should be guided by a thorough assessment of the specific needs, objectives, and operational conditions of the robotic welding industry. Implementing an effective FMEA can significantly improve maintenance management by systematically identifying potential failure points and applying proactive measures to mitigate risks. The study identified the following key benefits of an effective FMEA in Maintenance Management:

- Reduction of Unscheduled Downtime: Unexpected failures are reduced with a structured FMEA, increasing operational effectiveness and productivity. The firm was able to identify and rank the risks related to its maintenance procedures by using the Pareto Analysis, which ensured proactive risk management.
- Optimization of Maintenance Strategies: Better resource allocation and cost savings result from its improvement of maintenance schedules and procedures. To establish the frequency of PM and efficiently reduce the risks of downtime, an FMEA analysing the most frequent failures was developed.
- Enhanced Safety Measures: Through the identification of possible safety risks in maintenance tasks, FMEA guarantees that the appropriate safety measures are implemented to safeguard employees.
- Data-Driven Decision-Making: Senior management may better justify resource allocations and make well-informed decisions with the use of FMEA information.
- Continuous Improvement: Uptime and cost efficiency are increased when maintenance managers can monitor equipment performance, spot emerging failure risks, and enhance maintenance tactics through frequent updates to the FMEA.

In the future, investigating how machine learning models (such as anomaly detection and predictive analytics) can be trained on historical data to predict robotic welding system failures and assess how well AI-based predictive maintenance performs in comparison to conventional RCM techniques can be the focus of future research. Investigating the use of edge computing and the Internet of Things to process data closer to the machine for quicker decision-making. Researching the effects of fixed versus dynamic maintenance intervals on production costs and uptime is possible. Additionally, maintenance can be adaptively scheduled using research optimization methods (such as genetic algorithms and reinforcement learning) that consider factors like production demand, equipment condition, and failure likelihood.

By adopting a structured and proactive maintenance approach, organizations can effectively manage emerging issues, reduce failure recurrence, and ensure long-term reliability in robotic welding operations. Future research should focus on integrating advanced technologies to further optimize maintenance strategies and enhance industrial efficiency.

## Data Availability

The datasets presented in this article are not readily available due to time and technical constraints. Requests to access these datasets should be directed to the corresponding author.

## References

[B19] Accessed SAE JA1011 (2022). What asset managers need to know. Available online at: https://www.upkeep.com/learning/sae-ja1011 October 15, 2022).

[B1] Ahmad M. Benson R. (2007). Benchmarking in the process industry. UK: Institution of Chemical Engineers. Available online at: https://citeseerx.ist.psu.edu/viewdoc/download?doi=10.1.1.193.1196&rep=rep1&type=pdf.

[B2] Anton M. Yoshino K. (2003). Disney ride upkeep assailed. Los Angeles: Times. Available online at: https://Disney Ride Upkeep Assailed-Los Angeles Times (Accessed November 9, 2003).

[B3] Atikpakpa A. A. Okafor C. E. Okonkwo U. C. (2016). Failure and reliability evaluation of Turbines used in Nigerian Thermal plant. J. Sci. Technol. Environ. Inf. 04 (01), 280–292. 10.18801/jstei.040116.31

[B4] Bugaja M. Urminskýa T. Rostáša J. Pechoa P. (2019). Aircraft maintenance reserves – a new optimization approach. Transp. Res. Procedia 43, 31–40. 10.1016/j.trpro.2019.12.016

[B5] Curilem M. Araya B. Garrido F. Cubillos F. (2014). “Predictive models applied to Heavy duty equipment management,” in Mexican International Conference on Artificial Intelligence (Cham: Springer), 198–205.

[B6] El-Thalji I. Liyanage J. P. (2012). On the operation and maintenance practices of Wind Power assets: a status review and Observations. J. Qual. Maintenance Eng. 18 (3), 232–266. 10.1108/13552511211265785

[B7] Fore S. Msipha A. (2010). Preventive maintenance using reliability Centred maintenance (RCM): a case study of a Ferrochrome manufacturing company. South Afr. J. Industrial Eng. 21 (1), 207–235. 10.7166/21-1-78

[B8] Fouché H. J. (2015). Practical development of reliability-centered maintenance principles and practices: a hot strip mill as case study Supervisor.

[B9] Ghemari Z. Belkhiri S. Bezaf Y. Hamach A. Saad S. (2024). Application of the FMECA method for optimizing the reliability of the 1600T press. J. Adv. Manuf. Syst. 23 (03), 659–682. 10.1142/s0219686724500288

[B10] Halle C. (2019). Welding robots are on the rise: what’s your strategy? Evaluate risks and select the right safeguarding systems. Industrial Saf. and Hyg. News. Available online at: https://www.ishn.com/articles/110546-welding-robots-are-on-the-rise.

[B21] Kolte T. S. Dabade U. A. (2017). Machine operational availability improvement by implementing effective preventive maintenance strategies - a review and case study. Int. J. Eng. Res. Sci. Technol. 10, 700–708.

[B11] Montgomery D. C. Peck E. A. Vining G. G. (2021). Introduction to linear regression analysis. John Wiley and Sons.

[B12] MSG-3 (2018). Operator/Manufacturer Scheduled Maintenance Development (Vol. 1 – Fixed Wing Aircraft and Vol. 2 – Rotorcraft). United States: Airlines for America.

[B13] Nahatai T. Weenakorn I. Thanapong I. Watcharin K. (2023). On the Pulling linear regression and its applications in Digital Mammograms. WSEAS Trans. Inf. Sci. Appl. 20, 66–75. 10.37394/23209.2023.20.9

[B14] Okwuobi S. Ishola F. Ajayi O. Salawu E. Aworinde A. Olatunji O. (2018). A reliability-centered maintenance study for an individual Section-Forming machine. Machines 6, 50. 10.3390/machines6040050

[B15] Pancholi N. Bhatt M. (2018). FMECA-based maintenance planning through COPRAS-G and PSI. J. Qual. Maintenance Eng. 24, 224–243. 10.1108/JQME-03-2017-0015

[B16] Paolella M. S. (2018). Linear models and time-series analysis: regression. ANOVA, ARMA, and GARCH. John Wiley and Sons.

[B17] Ramli R. Arffin M. Z. (2012). Reliability centered maintenance in schedule improvement of automotive assembly industry. Am. J. Appl. Sci. 9 (8), 1232–1236. 10.3844/ajassp.2012.1232.1236

[B18] Refinery M. Dharmaraj A. (2019). Development of reliability centered maintenance in process industry – issues and difficulties.

[B23] Suryono M. A. E. Rosyidi C. N. (2018). Reliability centred maintenance (RCM) analysis of laser machine in filling lithos at PT X. IOP Conf. Ser.: Mater. Sci. Eng. 319 (1), 1757–8981. 10.1088/1757-899X/319/1/012020

[B20] Yavuz O. Doğan E. Carus E. Görgülü A. (2019). Reliability centered maintenance practices in Food industry. Procedia Comput. Sci. 158, 227–234. 10.1016/j.procs.2019.09.046

[B22] Zeinalnezhad M. Chofreh A. G. Goni F. A. Klemeš J. J. (2020). Air pollutionprediction using semi-experimental regression model and ddaptive neuro-fuzzy inference system. J. Clean. Prod. 261, 121218.

